# A System to Enrich for Primitive Streak-Derivatives, Definitive Endoderm and Mesoderm, from Pluripotent Cells in Culture

**DOI:** 10.1371/journal.pone.0038645

**Published:** 2012-06-11

**Authors:** Svetlana Vassilieva, Hwee Ngee Goh, Kevin X. Lau, James N. Hughes, Mary Familari, Peter D. Rathjen, Joy Rathjen

**Affiliations:** 1 School of Molecular and Biomedical Science, University of Adelaide, Adelaide, South Australia, Australia; 2 Department of Zoology, University of Melbourne, Parkville, Victoria, Australia; 3 The Menzies Research Institute Tasmania, University of Tasmania, Hobart, Tasmania, Australia; Instituto de Medicina Molecular, Portugal

## Abstract

Two lineages of endoderm develop during mammalian embryogenesis, the primitive endoderm in the pre-implantation blastocyst and the definitive endoderm at gastrulation. This complexity of endoderm cell populations is mirrored during pluripotent cell differentiation *in vitro* and has hindered the identification and purification of the definitive endoderm for use as a substrate for further differentiation. The aggregation and differentiation of early primitive ectoderm-like (EPL) cells, resulting in the formation of EPL-cell derived embryoid bodies (EPLEBs), is a model of gastrulation that progresses through the sequential formation of primitive streak-like intermediates to nascent mesoderm and more differentiated mesoderm populations. EPL cell-derived EBs have been further analysed for the formation of definitive endoderm by detailed morphological studies, gene expression and a protein uptake assay. In comparison to embryoid bodies derived from ES cells, which form primitive and definitive endoderm, the endoderm compartment of embryoid bodies formed from EPL cells was comprised almost exclusively of definitive endoderm. Definitive endoderm was defined as a population of squamous cells that expressed *Sox17,* CXCR4 and *Trh*, which formed without the prior formation of primitive endoderm and was unable to endocytose horseradish peroxidase from the medium. Definitive endoderm formed in EPLEBs provides a substrate for further differentiation into specific endoderm lineages; these lineages can be used as research tools for understanding the mechanisms controlling lineage establishment and the nature of the transient intermediates formed. The similarity between mouse EPL cells and human ES cells suggests EPLEBs can be used as a model system for the development of technologies to enrich for the formation of human ES cell-derived definitive endoderm in the future.

## Introduction

Endoderm is first observed in the mammalian embryo as a layer of primitive endoderm that forms across the exposed surface of the inner cell mass (ICM) from the *Gata6*-expressing cells within the ICM [1]. This population differentiates to give extraembryonic endoderm populations: visceral endoderm, which forms from cells that remain in contact with the pluripotent cells, and parietal endoderm, which forms from cells that migrate over the inner surface of the trophectoderm [2], [3]. A second endoderm lineage, the definitive or embryonic endoderm, arises at gastrulation [4]. Gastrulation initiates with the formation of the primitive streak, a region characterised by the localised breakdown of extracellular matrix and heralded by the expression of *Wnt3* and nuclear translocation of ß-catenin in a small population of cells at the prospective posterior embryonic-extraembryonic boundary [5], [6]. As embryogenesis proceeds the streak extends anteriorly towards the distal tip of the embryo. At the streak cells delaminate from the epiblast, differentiate and migrate between the ectoderm and visceral endoderm, giving rise to the mesoderm. These cells undergo an epithelial to mesenchymal transition and down regulate E-cadherin. Alternatively, cells traverse the primitive streak and intercalate with the adjacent visceral endoderm [7], after which they expand anteriorly and proximally, dispersing and displacing proximally the existing visceral endoderm such that the tissue layer that is traditionally referred to as definitive endoderm appears to comprise a mixed population of definitive endoderm and residual visceral endoderm [4], [8]. Cells fated to form definitive endoderm maintain expression of *E-cadherin* [9]. Definitive endoderm formed at gastrulation is the progenitor population of the gut tube and associated visceral organ derivatives.

Embryonic stem (ES) cells [10], [11], maintain many of the properties of the pluripotent cells of the ICM/pre-implantation epiblast, including the ability to differentiate into the three primary germ layers, the primitive endoderm and the primordial germ cell lineage (reviewed in [12], [13]). These cells form the basis of the pluripotent lineage which undergoes proliferation, differentiation and rearrangement to form a pseudostratified epithelium of pluripotent cells which lines the egg cylinder of the pre-gastrulation mouse embryo; this tissue has been referred to as primitive ectoderm or early post-implantation epiblast [13], [14]. In this report we will use pre-implantation epiblast to refer to the epiblast cells of the blastocyst and progenitors of ES cells, early primitive ectoderm to refer to the epiblast of the immediate post-implantation embryo at 5–5.5 d.p.c.[15] and late primitive ectoderm to refer to the pluripotent cells of the pre-gastrula and gastrula (5.5–7.5 d.p.c.) (also known as the late post-implantation epiblast [13]), reflecting three distinct stages of development in the pluripotent lineage [13], [15], [16].

The initial differentiation event in the development of the pluripotent lineage, the formation of early primitive ectoderm from the pre-implantation epiblast, can be recapitulated in culture with formation of early primitive ectoderm-like (EPL) cells from ES cells in response to factors within medium conditioned by HepG2 cells (MEDII) [17]. EPL cells share many properties with the early primitive ectoderm, including gene expression, cytokine responsiveness and differentiation potential [15], [17], [18], [19]. EPL cells also share properties with EpiSCs, cells derived from the late primitive ectoderm (from embryos between 5.5–6.5 d.p.c.), including morphology, increased expression of early and late primitive ectoderm markers *Fgf5* and *Otx2* when compared to mouse ES cells, and a differentiation potential that encompasses the three primary germ layers [20], [21], [22]. There are, however, significant differences between these populations. EpiSCs express *Nanog* at levels equivalent to mouse ES cells [20], [21] whereas *Nanog* expression is down regulated with EPL cell formation [22]. *Nanog* expression is lost with primitive ectoderm formation and is re-expressed in the late primitive ectoderm prior to gastrulation [23], [24], [25] suggesting that EPL cells represent Nanog^low^, early primitive ectoderm and EpiSC the *Nanog*-expressing late primitive ectoderm. A similar conclusion has been drawn from the comparison of the chromatin configuration of EPL cells and EpiScs, which suggests these cells represent populations that occur on either side of a global genome reorganisation, or autosomal lyonisation, with EPL cells representing early primitive ectoderm and EpiSC having their origin in the late primitive ectoderm [16]. In a recent review of pluripotent stem cells that discussed the transitional states that occurred within the pluripotent lineage EPL cells were defined as an distinct intermediary between ES cells and EpiSCs and representative of the early post-implantation epiblast [13].

Formation and differentiation of EPL cells in aggregates cultured in the presence of MEDII (EBM) results in cell populations restricted to the ectoderm, without the formation of visceral endoderm or mesoderm [26], demonstrating the ability of EPL cell differentiation to be directed to specific cell fates. The definitive endoderm is a key target of in vitro differentiation as it acts as the progenitor for a number of cell populations with projected clinical applications, most notably insulin-producing cells and hepatocytes. The unequivocal identification of definitive endoderm during in vitro differentiation, and discrimination of this population from the contemporaneous visceral endoderm, has proven difficult due to a paucity of specific definitive endoderm markers. Differentiation of EPL cells as embryoid bodies (EPLEBs) results in formation of aggregates enriched in mesoderm and largely devoid of ectoderm and visceral endoderm ([18] and data presented here). Characterising the progression of differentiation in EPLEBs shows the formation of primitive streak intermediates followed by the emergence of cells characteristic of mesoderm and endoderm, suggesting that differentiation within these bodies models differentiation that occurs in the posterior midline of the gastrulating mouse embryo. Using morphological comparison with the endoderm populations of pregastrula and gastrulating mouse embryo, gene expression and a functional protein uptake assay we have shown that the outer layer of cells in EPLEBs comprises a layer of definitive endoderm encapsulating an inner population of mesoderm. We propose that EPLEBs, which provide a novel source of nascent definitive endoderm in the near absence of contaminating visceral endoderm, will have applications in the development of protocols for cell differentiation and formation of later endoderm populations. Moreover, the recapitulation of the primitive streak in the absence of ectoderm lineages and contaminating visceral endoderm provides a model that can be used to characterise the regulatory mechanisms controlling cell differentiation and lineage choice within the primitive streak.

## Results

### Differentiation within EPLEBs recapitulates the processes occurring in the primitive streak

**Figure 1:**
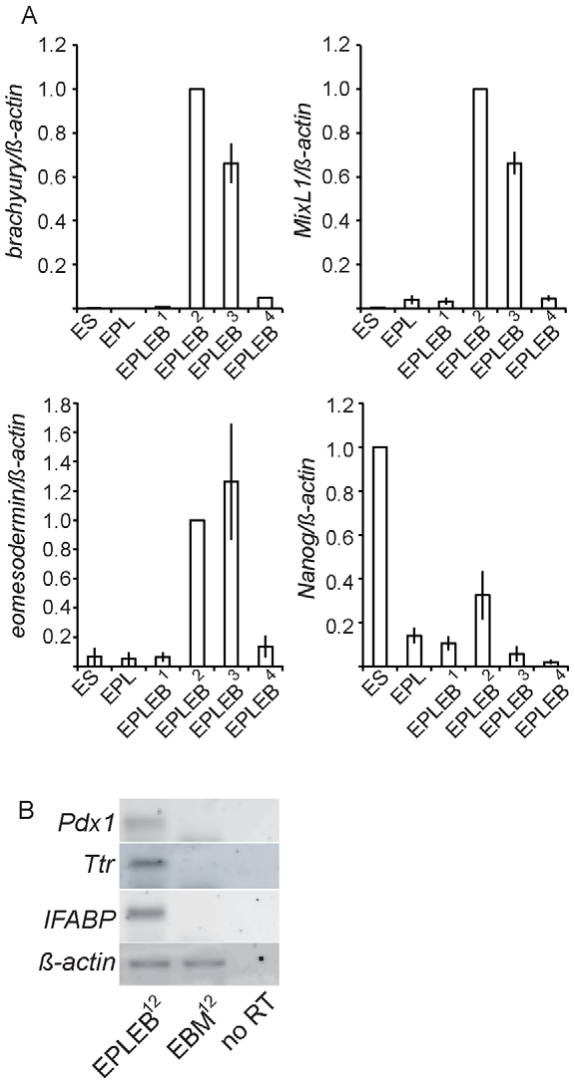
Posterior primitive ectoderm and primitive streak markers are expressed in differentiating EPL cells. **A.** q PCR analysis of RNA isolated from ES cells, EPL cells cultured for 2 days in MEDII (EPL) and EPLEBs formed from EPL cells and cultured for 4 days for the expression of *Brachyury, Mixl1*, *Eomesodermin*, and *Nanog*. Gene expression has been normalised to *actin* and is expressed relative to EPLEB^2^ (*Brachyury, Mixl1*, *Eomesodermin*) or ES cells (*Nanog*). n = 3. Error bars represent standard error of the mean. **B.** RNA was extracted from EPLEBs and EBMs on day 12 and analysed by RT-PCR for the expression of a number of genes characteristic of definitive endoderm cell lineages. Reactions in which reverse transcriptase has been omitted (no RT) were included as controls.

Differentiation in EPLEBs, when compared to EBs, is characterised by the earlier expression of *Brachyury*, a marker of the primitive streak intermediate and early mesoderm, on days 2–3 compared to days 4–5 [18]. Earlier differentiation within EPLEBs is consistent with the prior formation of EPL cells from ES cells. EPLEBs were analysed for the expression of additional primitive streak intermediate markers, *Eomesodermin* [27] and *Mixl1* [28] (Figure 1A). Both genes showed a pattern of expression in EPLEBs equivalent to *Brachyury*. *In vivo, Nanog* expression is detected in the ICM [23], [24], down regulated in late blastocysts and up regulated in posterior primitive ectoderm prior to gastrulation [25]; expression is lost as cells ingress through the streak [23], [25]. During the formation and differentiation of EPL cells, *Nanog* expression was initially decreased with the generation of EPL cells (Figure 1A), transiently increased on day 2 of differentiation in EPLEBs, coincident with the onset of primitive streak marker expression (Figure 1A), before being lost. The temporally restricted expression of *Brachyury, Mixl1* and *Eomesodermin* on days 2 and 3 suggests differentiation of EPL cells in EPLEBs to the primitive streak intermediate is relatively synchronous and occurs within a 48 hour window.

The primitive streak gives rise to both mesoderm and definitive endoderm progenitors; the cell populations formed within EPLEBs have been shown previously to comprise terminally differentiated mesoderm derivatives [18]. The expression of markers of endoderm-derived populations in EPLEBs was determined by RT-PCR on day 12 of differentiation and compared to expression in EBM^12^, a population of cells comprised exclusively of neural ectoderm and neural ectoderm derivatives, and devoid of definitive endoderm [26]. EPLEBs, but not EBMs, expressed *Pdx1*, a gene expressed in the progenitors of the pancreas, stomach and duodenum [29], [30], *Intestinal Fatty Acid Binding Protein* (*IFABP*), an early posterior gut marker [31] and *Transthyretin* (*Ttr*), a gene expressed in the early and mature hepatocyte lineages [32], suggesting the formation of differentiated endoderm populations within these bodies (Figure 1B).

### The morphology of the outer cell layer of EPLEBs is consistent with formation of definitive endoderm

Morphology has been used as a criterion for distinguishing populations of visceral and parietal endoderm in the embryo [33] and in embryoid bodies [34], [35], [36], [37]. Here, a detailed morphological examination was undertaken to identify and classify the endoderm of EPLEBs, EBs and EBMs.

**Figure 2:**
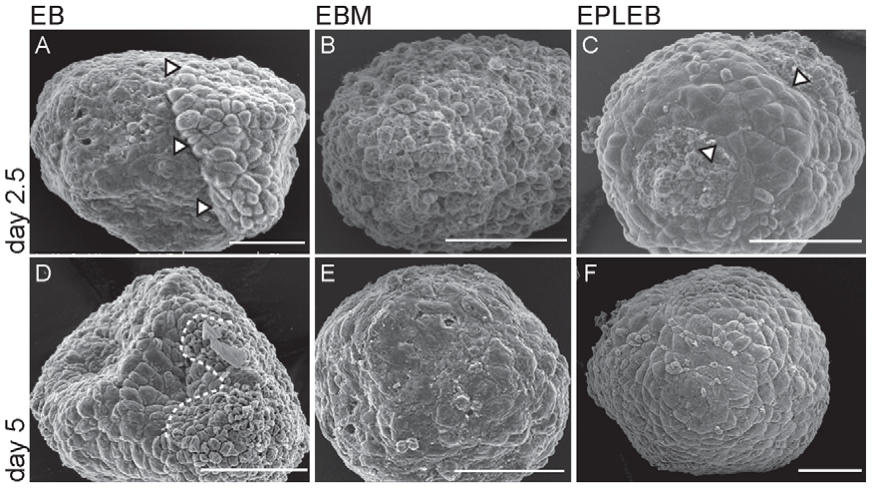
Formation of endoderm on EBs, EPLEBs, EBMs. **A–F.** Scanning electron micrographs of EBs (A, D), EBMs (B, E) and EPLEBs (C, F) on days 2.5 (A–C) and 5 (D–F). Arrowheads mark the boundary of the prospective patch of primitive endoderm on EBs on day 2.5 (A) and the prospective endoderm population forming on the surface of EPLEBs at 2.5 days of differentiation (C). The dotted line on D demarcates the boundary of two distinct surface morphologies. Size bars represent 50 μm.

Scanning electron microscopy (SEM) was used to characterise the surface morphology of EBs, EBMs and EPLEBs. On day 2.5 the surface of EBs was characterised by the presence of patches of rounded cells (Figure 2A, patch delineated by arrowheads). By day 5 the EBs had a surface morphology which appeared to be comprised of several distinct cell types, most notably a population of small, loosely adherent rounded cells and a population of larger, squamous cell (Figure 2D; the right and left side of the aggregate shown, respectively). The surface morphology of EBMs was very different. On day 2.5 no obvious patches of rounded cells could be seen and the surface of the body was uniformly granular (Figure 2B). On day 5 the surface of EBMs was compacted, with no distinct cell morphology or cell junctions able to be discerned (Figure 2E). This, coupled with the lack of endoderm-specific gene expression (Figure 1B and [26]) seen in these bodies suggests that no outer layer of endoderm is formed in EBMs. EPLEBs presented a third, distinct surface morphology. On day 2.5 patches of cells with a flattened morphology were present on the surface of EPLEBs (Figure 2C, population is bracketed with arrowheads). By day 5 cells of this morphology comprised the entire outer layer of EPLEBs (Figure 2F). The characteristic surface morphology of EPLEBs was present in 90% of the EPLEBs within the population.

**Figure 3:**
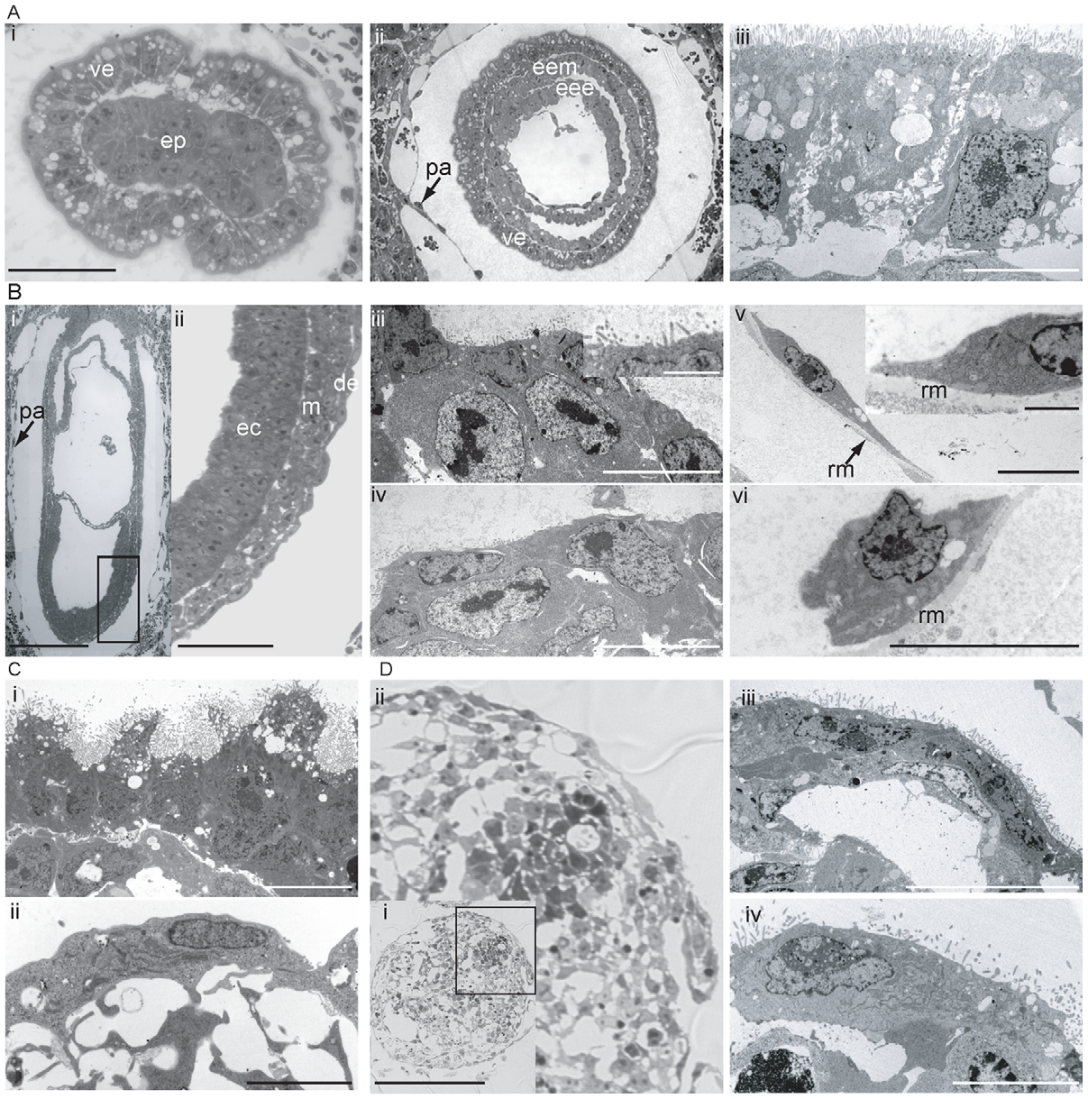
Characterisation of the endoderm populations seen in EBs and EPLEBs by comparison with the endoderm populations of the 6.5 and 7.5 d.p.c. embryo. **A.** (**i**) A 1 μm transverse section across the distal tip of a 6.5 d.p.c mouse embryo showing the distinctive morphology of the visceral endoderm (ve) surrounding the inner pluripotent cell core (ep). Size bar represents 40 μm. (**ii**) 1 μm transverse section across the extraembryonic region of a 7.5 d.p.c embryo showing the visceral endoderm (ve) surrounding the extraembryonic mesoderm (eem) and extraembryonic ectoderm (eee). Parietal endoderm (pa) is indicated by an arrow. (**iii**) Transmission electron micrographs (TEM) of visceral endoderm from the extraembryonic region of a 7.5 d.p.c. embryo, showing the typical cuboidal cell morphology with large apical vacuoles and dense microvilli on the apical surface, which can be seen at the top of the figure. Size bar represents 10 μm. **B.** (**i**) Longitudinal section of a 7.5 d.p.c. late-streak stage embryo. Size bar represents 200 μm, posterior to the right, parietal endoderm (pa) indicated by an arrow. (**ii**) Detail of (i), showing the trilaminar structure of the egg cylinder, with an outer layer of definitive endoderm (de), middle layer of mesoderm (m) and inner layer of ectoderm (ec) Size bar represents 40 μm. (**iii, iv**) TEM of definitive endoderm, showing an outer, squamous, cell layer of endoderm with a sparse decoration of microvilli on the apical surface. Inset shows the surface of the cells at a higher magnification. Size bars represent 10 μm (inset 2 μm). (**v, vi**) TEM of parietal endoderm, showing a dispersed, squamous, cell population in close contact with Reichart's membrane (rm), indicated by an arrow. The surface of the parietal endoderm is devoid of microvilli. Inset shows the surface of the cells at a higher magnification. Size bars represent 10 μm (inset 2 μm). **C.** (**i, ii**) TEM of the surface populations of cells observed on a day 5 EB. Cells appear reminiscent of the visceral (**i**) and parietal (**ii**) endoderm populations of the embryo. Size bars represent 10 μm. **D**. (**i**) Toluidine blue-stained 1 μm section of an EPLEB at day 5 of differentiation. Size bar represents 200 μm. (**ii**) Detail of (**i**) showing the squamous outer cell layer. (**iii, iv**) TEM of the outer layer of cells of EPLEB on day 5 of differentiation, showing an outer, squamous, cell layer with sparse microvilli on the apical surface, reminiscent of the morphology of the embryonic definitive endoderm. Size bars represent 10 μm (**iii**) and 5 μm (**iv**).

To identify the cell population on the surface of EPLEBs and EBs, bodies on day 5 were compared to the endoderm of mouse embryos by TEM. Transverse sections of a pre-gastrula (6.5 d.p.c.) and gastrulating (7.5 d.p.c.) embryo show the distinctive morphology of visceral endoderm (Figure 3Ai, ii, iii), a population of cuboidal cells with large, apical vacuoles and densely decorated with microvilli on the apical surface. The morphology we see at 6.5 d.p.c. is distinct from the morphology of the endoderm shown by others [7], [38] at this time of development; this may reflect differences in the timing of embryos which is complicating comparison. In contrast, parietal endoderm consisted of squamous cells devoid of microvilli, enriched in rough endoplasmic reticulum and located distantly from the pluripotent cells (Figure 3Bi, v, vi) [33]. Longitudinal sections of a 7.5 d.p.c. embryo show a typical trilaminar structure (Figure 3Bi, ii) in the embryonic region with an outer layer of definitive endoderm that morphologically is distinct from visceral and parietal endoderm and spatially and temporally consistent with definitive endoderm. Cells within this layer were squamous, sparsely decorated on the apical surface by microvilli and devoid of large vacuoles (Figure 3Biii, iv). This layer appears morphologically uniform, suggesting that any residual visceral endoderm cells acquire a morphology typical of the definitive endoderm [8].

Sections through EBs cultured to day 5 showed a heterogeneous population of cells on the surface; cells reminiscent of visceral (Figure 3Ci) and parietal (Figure 3Cii) endoderm were observed. Sections through an EPLEB cultured to day 5 demonstrated an outer cell layer morphologically distinct from visceral and parietal endoderm populations and consistent with definitive endoderm of the 7.5 d.p.c. embryo (Figure 3Di–iv).

### Gene expression and functional assays define an outer population of definitive endoderm and an inner population of nascent mesoderm in EPLEBs

**Figure 4:**
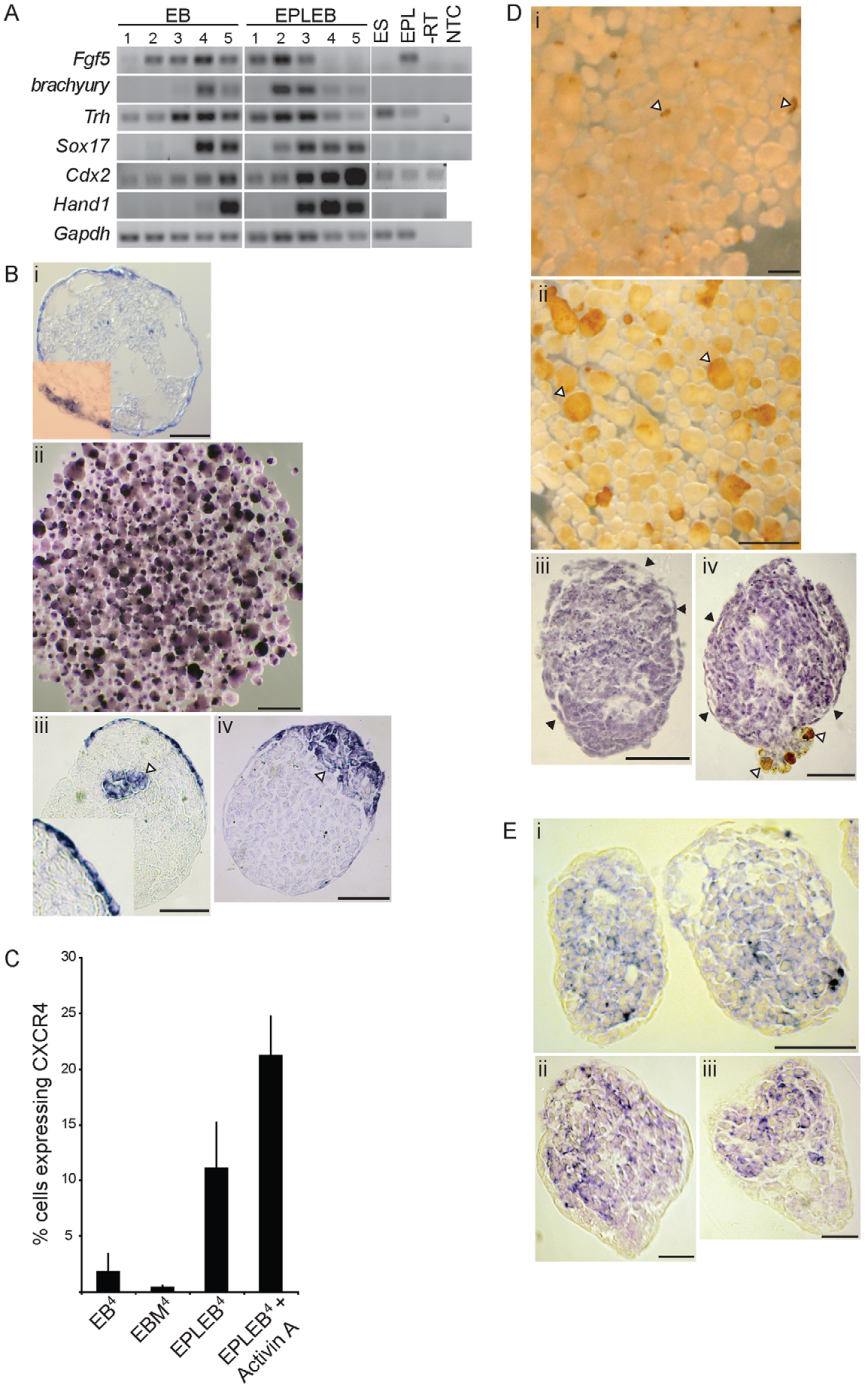
Localisation of definitive endoderm and mesoderm marker expression in EPLEBs. **A.** RNA isolated from EBs and EPLEBs on days 1–5 of differentiation was analysed by RT-PCR for the expression of *Fgf5, Brachyury, Trh, Sox17, Cdx2* and *Hand1. Gapdh* expression was used a loading control. Expression in undifferentiated ES cells (ES) and EPL cells (EPL) is shown for comparison. -RT (control, no reverse transcriptase) and a no template control (NTC) were included. n = 3, a representative result is shown. **B.** Wholemount *in situ* hybridisation of EPLEBs on day 5 of differentiation with a DIG-labelled probe complimentary to *Sox17* (**i**) or *Trh* (**ii, iii, iv**). Representative aggregates are shown sectioned into 10 µm slices. Size bars represent 50 µm (i, iii, iv) or 500 µm (ii). **C.** EBs, EBMs, EPLEBs and EPLEBs cultured in the presence of 30 ng/mL Activin A were analysed by flow cytometry for the presence of CXCR4 positive cells. n = 3. **D.** Low magnification image of EPLEBs (**i**) and EBs (**ii**) on day 5 and day 7 respectively, stained for the uptake of horse radish peroxidase (HRP). Open arrowheads indicate areas of staining. Representative EPLEBs with (**iii**) or without (**iv**) a foci of HRP activity are shown sectioned and counterstained with haematoxylin to illustrate cell morphology. Open arrowheads indicate areas of staining, closed arrowheads indicate the non-staining endoderm layer on the outside of the EPLEBs. Size bars represent 500 µm (i, ii,) or 50 µm (iii, iv). **E.** Wholemount *in situ* hybridisation of EPLEBs on day 5 of differentiation using a DIG-labelled probe complimentary to *Cdx2* (**i**) or *Hand1* (**ii, iii**). Representative aggregates are shown sectioned into 10 µm slices. Size bars represent 50 µm.

Definitive endoderm in the embryo can be characterised by ontogeny, through its origin from the primitive ectoderm during gastrulation, by expression of markers of endoderm, including the *Sry*-related HMG box gene, *Sox17* [39] and *Thyrotropin-releasing Hormone*, *Trh* [40], and by a horseradish peroxidase (HRP) uptake assay which identifies the neighbouring visceral endoderm through its ability to endocytose proteins from the surrounding medium [39]. Gene expression analysis of EBs and EPLEBs for *Fgf5*, which tracks the formation and differentiation of EPL cells, *Brachyury*, which marks differentiation to primitive streak intermediates, *Sox17*, which is expressed within the visceral and definitive endoderm [39] and *Trh*, which identifies the prospective definitive endoderm and which distinguishes visceral and definitive endoderm in the embryo [40], was performed by RT-PCR (Figure 4A). As expected, *Fgf5* was expressed in EPL cells and early EPLEBs, with the level of expression reducing on day 3, after the onset of differentiation on day 2 as determined by *Brachyury*. In EBs, in contrast, *Fgf5* expression was not initiated until 48 hours after ES cell aggregation and persisted beyond day 4, marking the formation of primitive ectoderm in these aggregates. *Sox17* expression was also detected earlier in EPLEBs when compared to EBs; up regulation of *Sox17* was coincident with differentiation, as determined by *Brachyury* expression, and with the observed formation of the outer layer of endoderm in EPLEBs (Figure 2). In EPLEBs *Sox17* expression was sustained to day 5, the limit of this assay. *Trh* expression was detected in ES cells and EPL cells and throughout early EPLEB differentiation, consistent with the expression of this gene in the primitive ectoderm and definitive endoderm [40]. Expression of both *Fgf5* and *Trh* in EPLEBs on days 1–3 marks the presence of primitive ectoderm; the subsequent decline in *Fgf5* expression and persistence of *Trh* expression, coupled with the up regulation of *Sox17* expression, is consistent with the differentiation of the primitive ectoderm and formation of definitive endoderm in these aggregates.

Wholemount *in situ* hybridisation detected *Sox17* and *Trh* transcripts within cells of the outer layer of EPLEBs on day 5 (Figure 4B). Higher magnification images show these cells to be morphologically consistent with a squamous cell type, consistent with the identification of definitive endoderm. *Trh* expression was also detected in inner cells of the aggregates (Figure 4Biii, iv; open arrowheads).

Expression of the chemokine receptor CXCR4 has been correlated with definitive, but not visceral, endoderm [41], [42]. The number of cells within EBs, EBMs and EPLEBs on day 4 expressing CXCR4 was determined by flow cytometry (Figure 4C). As expected, very few CXCR4 expressing cells were detected in EBMs or in EBs at this time point; this suggests that the endoderm populations seen on the surface of EBs early in differentiation were primitive and not definitive. In contrast, approximately 12% of cells expressed CXCR4 in EPLEBs on day 4. The formation of definitive endoderm during differentiation can be enhanced by with the addition of Activin A [43]; addition of 30 ng/mL Activin A to EPLEBs increased the proportion of cells expressing CXCR4.

Neither *Sox17* nor *Trh* were expressed in all the cells of the outer endoderm layer in EPLEBs suggesting a degree of cell heterogeneity. To ensure that non-expressing cells within the layer were not visceral endoderm, an HRP uptake assay was undertaken. Cells of the visceral endoderm will take up HRP from the surrounding medium and stain dark brown when developed with DAB. The majority of EPLEBs lacked areas of staining on their surface (Figure 4Di, iii; filled arrowheads), consistent with the endoderm layer comprising definitive, and not visceral, endoderm. Rare EPLEBs had small areas of stained cells (Figure 4Di and iv, open arrowheads); closer examination of these cells showed that they were large, bubbly, cuboidal cells, morphologically distinct from the majority of the cells on the surface of the aggregates and consistent in appearance and properties with visceral endodermal cells. DAB-staining cells were more prevalent in EBs (Figure 4Dii).

The outer cells of EPLEBs are comparable to definitive endoderm by morphology, gene expression and function. By analogy with the primitive streak, which gives rise to definitive endoderm and mesoderm, we would propose that the cells located internally to the endoderm in EPLEBs would express markers of mesoderm. To define mesoderm in EPLEBs we selected and validated two genes from a microarray of EPL cells compared to EPLEBs on day 2 and 4; *Cdx2* and *Hand1* were expressed in EPLEBs on day 4 in the microarray, after markers of the primitive streak intermediate and in later mesoderm tissues of the mouse embryo, (KXL and JR unpublished). *Cdx2* is expressed in the primitive streak of 7.5 d.p.c. embryos [44] whereas *Hand1* is expressed in neural crest derivatives and lateral mesoderm [45], [46]. Recently, CDX2 has been shown to be co-expressed with BRA in mesoderm formed from human ES cells in response to BMP4 [47]. As expected, *Cdx2* and *Hand1* were expressed in EPLEBs, and expression initiated earlier when compared to EBs (Figure 4A). The up regulation of both genes lags the expression of *Brachyury* and is consistent with expression in mesoderm derivatives in the aggregates. Wholemount *in situ* hybridisation detected *Cdx2* (Figure 4Ei) and *Hand1* ((Figure 4Eii, iii) transcripts in cells within the EPLEBs and not in cells within the outer endoderm cell layer.

## Discussion

### Definitive endoderm is formed in EPLEBs

Differentiation of pluripotent cells during gastrulation *in vivo* and pluripotent cell differentiation *in vitro* results in similar cellular outcomes but the ability to demonstrate these outcomes is often hampered by a non-specificity of markers and a lack of spatial and temporal information in culture. Many genes that have been identified as definitive endoderm markers in the embryo, such as *Ihh*, *Gata4, Gata6, Sox17, Cxcr4* and *Foxa2*, are expressed in multiple tissues [35], [48], [49], [50], [51], [52]. *Sox17*, for example, is expressed within both the visceral endoderm, adjacent to the extraembryonic ectoderm, and in the definitive endoderm [39]. The formation of visceral and definitive endoderm in EBs [37], [43], [53], a system which lacks the spatial organisation of the embryo, has hampered unequivocal identification of endoderm populations by marker analysis. Coupling detailed morphological analysis, using SEM and TEM, with the expression of *Sox17* and *Trh* and the functional HRP uptake assay has allowed unambiguous identification of definitive endoderm in EPLEBs; by morphology the endoderm compartment on over 90% of the EPLEBs comprised definitive endoderm with little or no visceral endoderm observed in the populations. Moreover, visceral endoderm represented a small component of the overall endoderm population in EPLEBs containing foci of HRP uptake. This is in contrast to EBs which formed visceral and definitive endoderm as shown by morphology and HRP uptake. The prevalence of definitive endoderm in EPLEBs, 12% of total cells on day 4, compares favourably with other systems use for the derivation of this cell type [54], [55]. Furthermore, the ability to further enrich for the population with Activin A, to approximately 20% of total cell number, makes EPLEBs an attractive system for deriving definitive endoderm in culture and one that could be integrated into existing methodologies for enrichment of definitive endoderm from ES cells [42], [56].

Unlike the pluripotent cells of the ICM, the primitive ectoderm in the embryo is thought to be limited in its ability to give rise to the primitive endoderm lineage [14], [57]; this could be a consequence of a loss of developmental potential when compared to the pre-implantation epiblast such that the primitive ectoderm is no longer capable of giving rise to the primitive endoderm lineage, or it could be a constraint imposed on the primitive ectoderm by the local environment. When compared to EBs, EPLEBs showed a reduction in primitive endoderm formation when differentiated within embryoid bodies. HRP uptake assays revealed only small areas of visceral endoderm on the surface of occasional EPLEBs; these areas have a frequency and morphology equivalent to the occasional areas of AFP^+^ cells that have been detected previously [18]. This contrasts with the more extensive formation of visceral endoderm on EBs revealed by HRP uptake. We have reported previously the formation of parietal endoderm on the surface of EPLEBs [18]. In this study we were unable to detect parietal endoderm by morphology or by *SPARC* expression (JR, unpublished), and suggest that this endoderm population, like visceral endoderm, occurs at very low levels and sporadically within populations of EPLEBs.

The morphological characterisation of EPLEBs showed the definitive endoderm arranged as a continuous or near continuous layer of cells on the outer surface of EPLEBs. In the embryo, cells forming definitive endoderm traverse the anterior primitive streak and intercalate into the adjacent visceral endoderm [4], with visceral endoderm potentially guiding the organisation of the definitive endoderm. In EBs, where endoderm is similarly organised as an outer layer on the aggregates the initial formation of a layer of primitive/visceral endoderm could act as a guide for the organisation of the emerging definitive endoderm. In EPLEBs the organisation of the definitive endoderm occurs, however, in the absence of an existing primitive endoderm layer and suggests that cell organisation is cell autonomous and similar to the ability of the primitive endoderm to organise as a cell layer during differentiation within EBs [58]. *In situ* localisation of *Trh* revealed foci of internally located *Trh^+^* cells; these foci may contain residual pluripotent cells [40] or early progenitors of the endoderm. The majority of these foci were located subjacent to the outer endoderm layer consistent with a differentiation process that adds cells to the outer surface of the cell aggregate.

The difficulty in unambiguously identifying endoderm populations has meant that few ES cell differentiation studies have evaluated the relative frequency of primitive endoderm derivatives, particularly visceral endoderm, and definitive endoderm in the differentiated cell population. Yasanuga et al. [42] and D'Amour et al. [56] preferentially enriched for definitive endoderm by addition of Activin A to differentiating cells in culture; in both cases it was demonstrated that this effectively reduced the contribution to the population by the visceral endoderm. Morrison et al. [54] also used Activin A to enrich for definitive endoderm from ES cell. They described a role for FGF signalling, in combination with Activin A, in the specification of an endoderm subpopulation, the anterior definitive endoderm. We have been unable to detect *Hex^+^* cells in EPLEBs by *in situ* hybridisation (data not shown) suggesting that anterior definitive endoderm is not formed in this system. This is perhaps not surprising given the requirement for FGF signalling in the specification of this population [54]. In comparison to these growth factor-based methodologies, the enrichment of definitive endoderm, but not primitive endoderm, in EPLEBs is achieved by manipulating the pluripotent state of the starting cell population and using differentiation conditions that enforce the formation of the primitive streak intermediate. Directing the differentiation of EPL cells to primitive streak intermediates is achieved by removing visceral endoderm-like signalling from the medium, present in this system in the conditioned medium, MEDII, and by disrupting cell:cell associations [59]. The ability to form definitive endoderm, in the effective absence of visceral endoderm, and without the use of growth factors, provides a viable alternate methodology for achieving a population of definitive endoderm that can be used for further differentiation and formation of later endoderm populations, and a differentiation system which can be easily manipulated through the addition of exogenous growth factors.

### Differentiation of EPL cell: a model for differentiation on the posterior side of the gastrulating mouse embryo

In the embryo, formation of the mesoderm and definitive endoderm is restricted to the posterior of the embryo whereas ectoderm forms from cells at the anterior [7]. The lack of axes and temporal restrictions on differentiation during EB differentiation, however, generally results in an unstructured cell mass in which inappropriate signalling and cell:cell interaction can occur. This dysregulation can confound the study of differentiation *in vitro* [60] and does result in heterogeneous cellular outcomes.

Using EPL cells as starting material for *in vitro* differentiation appears to overcome a number of these inherent difficulties. As reported previously, and further demonstrated here, EPL cell differentiation results in germ layer formation without the initial formation of the primitive and visceral endoderm [18], [26]. Any initial requirement for visceral endoderm signalling in the loss of pluripotence and formation of mesoderm and definitive endoderm is replaced in this system by the disruption of cell interactions and removal of MEDII [59]. In the embryo, later populations are specified through interaction of the germ lineages; this occurs after the primitive endoderm lineages have been dispersed and displaced proximally. Close interactions between the germ lineages and the visceral endoderm do not appear to be required although the role of the dispersed, *Ttr^+^* cells in the endoderm layer has not been understood [8]. This is largely recapitulated in EPLEBS, where further differentiation occurs without visceral endoderm signalling. The embryonic environment is more poorly recapitulated in EBs where later lineage specification occurs in an environment comprising the germ lineages and the primitive endoderm. The consequences of the complicated signalling environment in EBs are not known.

EPLEBs model the gastrulation events that occur in the primitive streak and differentiation results in a simple cell aggregate spatially organised into an outer layer of definitive endoderm and an inner parenchyma comprised largely of mesoderm. The relative simplicity of this differentiation system allows analysis of the molecular and cellular events of the primitive streak [22], [59], [61], including characterisation of the role of exogenously added signalling molecules in the processes of pluripotent cell differentiation, without the confounding influence of endogenous signalling from the primitive endoderm or the inappropriate juxtaposition of cell populations. Furthermore, this system is ideal for future work into understanding the role of cell interactions in later differentiation.

The formation of multiple endoderm populations during ES cell differentiation in culture as shown here and by others, and the difficulties in separating the endoderm populations using marker gene expression, has hindered the purification of the definitive endoderm for use as a substrate for further differentiation and led to suggestions that the origin of definitive endoderm-derived populations formed from ES cells may, in fact, be an extraembryonic lineage [42]. The use of EPL cells as a starting point for differentiation overcomes these limitations and provides a route to enrichment of definitive endoderm without the concomitant formation and elaboration of visceral endoderm or the need to use modified cell lines to facilitate cell-sorting.

## Materials and Methods

### Cell culture

D3 embryonic stem cells [62] (obtained from Lindsay Williams, Ludwig Research Institute, Melbourne) were maintained in the absence of feeders as previously described [17], [63]. EPL cells were formed as adherent cultures in medium supplemented with MEDII (50% MEDII) as previously described [18], [63]. Embryoid bodies (EB) were formed from ES cells and maintained as described in [18] and [63]. Alternatively, ES cells were differentiated by culturing for 2 or 3 days in 50% MEDII to form EPL cells before EB formation, giving rise to EPL cell-derived EBs (EPLEBs) [18], [63]. EBs cultured in MEDII-containing medium (EBMs) were formed from ES cells and maintained as described in [26] and [63].

### Scanning Electron Microscopy (SEM)

EBs, EBMs, and EPLEBs were fixed for 30 minutes in 4.0% paraformaldehyde/1.25% glutaraldehyde/PBS with 4% sucrose, pH 7.2, washed in PBS/4% sucrose and post-fixed in 2% osmium tetroxide (60 minutes). Samples were dehydrated (70%, 90%, 95% and 100% ethanol, 2×10 minutes each, 100% ethanol, 3×30 minutes) and dried in a Balzers CPD 030 critical point dryer (Principality of Liechtenstein), with CO_2_. Samples were mounted on stubs, coated with carbon and gold and examined at an accelerating voltage of 10 kV using a Philips XL20 scanning electron microscope (Phillips, The Netherlands).

### Transmission Electron Microscopy (TEM)

6.5 and 7.5 d.p.c. embryos, EBs and EPLEBs were fixed and dehydrated as for SEM before transfer to propylene oxide for 20 mins. Samples were infiltrated overnight with 1∶1 mixture of propylene oxide and epoxy resin before being infiltrated by100% resin, embedded and polymerised at 70°C for 24 hours. Embedded material was sectioned on an UltraCut E Ultramicrotome (Reichert-Jung, Austria) using a diamond knife (Diatome, Switzerland); 70 nm sections were cut. Sections were picked up on 200 mesh grids and stained for 10 minutes each with 4% saturated uranyl acetate and Reynold's lead citrate. Sections were viewed using the Philips CM100 transmission electron microscope (Phillips, The Netherlands).

### Gene expression analysis

PCR: Total RNA was isolated using RNAwiz (Ambion) or TRIzol® reagent (Invitrogen) and reverse-transcribed with oligodT (Invitrogen) using Omniscript RT kit (Qiagen) or M-MLV Reverse Transcriptase (Promega). PCR was performed as follows: 94°C for 3 minutes, the specified number of cycles of 94°C for 1 minute, 60°C for 30 seconds (annealing; 50°C *Actin*) and 72°C for 1 minute, followed by 7 minutes at 72°C. PCR products were separated on 2% agarose gels, stained with ethidium bromide or SYBR® Gold (Invitrogen) and detected using a BioRad FX imager (BioRad). Alternatively, real-time PCR was performed on a PCR thermal cycler (MJ Research) with a Chromo 4 Continuous Fluorescence Detector (MJ Research) using Platinum SYBR Green qPCR SuperMix UDG (Invitrogen) according to manufacturer's instructions. Expression levels were normalised using *β-actin*. Primer sequences, product sizes and cycle times are listed in Table 1.

**Table 1:** Primers used for RT-PCR and qRT-PCR analysis of gene expression.

Gene	Size	Primers (5′–3′)	Cycle
*Actin*	501	ATGGATGACGATATCGCTG	26
		ATGAGGTAGTCTGTCAGGT	
*Cdx2*	197	CCCTAGGAAGCCAAGTGAAAACC	36
		CTCCTTGGCTCTGCGGTTCTG	
*Fgf5*	169	CTGCAGATCTACCCGGATG	25
		TAAATTTGGCACTTGCATGG	
*Hand1*	101	GAAAGCAAGCGGAAAAGGGAG	32
		GGTGCGCCCTTTAATCCTCTT	
*Gapdh*	236	CTTCACCACCATGGAGAAGGC	18
		GGCATGGACTGTGGTCATGAG	
*IFABP*	225	GGAAAGGAGCTGATTGCTGTCC	39
		CTTTGACAAGGCTGGAGACCAG	
*Pdx1*	325	CCACCCCAGTTTACAAGCTC	39
		TGTAGGCAGTACGGGTCCTC	
*Sox17*	186	CTTTATGGTGTGGGCCAAAG	32
		TTGTAGTTGGGGTGGTCCTG	
*Trh*	163	CTGGAAGCAGCCCAGGAG	
		CCGGATGCTGGCGTTTTG	
*Ttr*	440	AGTCCTGGATGCTGTCCGAG	39
		TTCCTGAGCTGCTAACACGG	

In situ hybridisation: EPLEBs were fixed in 4% paraformaldehyde for 30 minutes at RT, and dehydrated in 50%, 75% and 100% methanol. *In situ* hybridisation was performed as described in [18]. Anti-sense DIG labelled probe for *Sox17* was transcribed from plasmid J8.1. Anti-sense and sense *Trh* riboprobes were synthesize as run-off transcripts from a pGEMT-easy vector (Promega) containing a 408 bp cDNA fragment linearised with NcoI and Sal1 using SP6 RNA polymerase and T7 RNA polymerase, respectively. Anti-sense and sense DIG labelled probes for *Cdx2* were transcribed from a pGEM-vector containing a 2.2 kbp *Cdx2* cDNA fragment that had been linearized with EcoRI or BamH1 and synthesized with SP6 RNA polymerase and T7 RNA polymerase respectively. Antisense and sense DIG labelled probes for *Hand1* probes were synthesized from a pBSK-vector (Thermo scientific) containing a 0.35 kbp *Hand1* cDNA fragment (from Prof. Richard Harvey, Victor Chang Research Institute, Sydney) that had been linearised with EcoRI and HindIII and synthesized with T7 or T3 RNA polymerase respectively.

### Horseradish peroxidase (HRP)-uptake assay

HRP-uptake assay was performed as previously described [39]. Briefly, cell aggregates were incubated in Dulbecco's modified Eagle medium containing 10% BSA and horseradish peroxidase (HRP; Sigma type VI, 2 mg/mL) for 30 minutes, after which aggregates were fixed in 4% PFA and developed with a 3,3′-diaminobenzidine (DAB; Sigma) in the presence of hydrogen peroxidase.

### Flow cytometry

Cell aggregates were trypsinised to a single cell suspension. 1×10^6^ cells were incubated with anti-CXCR4 antibody (Rat anti-mouse CD184 (CXCR4), 0.25 ng/mL, BD Biosciences) or an isotype control antibody for 45 minutes on ice. Cells were washed 3×5 minutes with PBS and incubated with a FITC-conjugated anti-rat secondary antibody (Jackson Laboratories). Cells were stained with 5 µL of a 5 µg/mL solution of propidium iodide for 5 minutes on ice before 3×5 minutes washes with PBS. Cell suspensions were analysed using a Becton Dickinson FACScan and data collected using CellQuest Pro software (BD) and manipulated using either CellQuest Pro or FCS Express (Microsoft). Dead cells were excluded using a size gate and PI staining. Background staining was determined using the isotype control and CXCR4 positive cells were plotted as a percentage of live cells.
